# Supplementary material for: LaCl3-based sodium halide solid electrolytes with high ionic conductivity for all-solid-state batteries
Source: Nat Commun. 2024 May 21;15:4315. doi: 10.1038/s41467-024-48712-4 (PMC11109254; doi:10.1038/s41467-024-48712-4)
Supplement: Supplementary file 1 — Supplementary Information [file 41467_2024_48712_MOESM1_ESM.pdf]

Supporting Information

$LaCl_3$-Based Sodium Halide Solid Electrolytes with High Ionic Conductivity for All-Solid-State Batteries

Chengyu Fu[1#], Yifan Li[2#], Wenjie Xu[3, 4#], Xuyong Feng[1, 5*], Weijian Gu[1], Jue Liu[6], Wenwen Deng[7], Wei Wang[8], AM Milinda Abeykoon[9], Laisuo Su[10], Lingyun Zhu[11], Xiaojun Wu[2], Hongfa Xiang[1, 5*]

[#] These authors contributed equally

[1] School of Materials Science and Engineering, Hefei University of Technology, Hefei 230009, Anhui, China

[2] School of Chemistry and Material Sciences, University of Science and Technology of China, Hefei, Anhui 230026, China

[3] National Synchrotron Radiation Laboratory, University of Science and Technology of China, Hefei, Anhui 230029, China

[4] School of Nuclear Science and Technology, University of Science and Technology of China, Hefei, Anhui 230029, China

[5] Engineering Research Center of High-Performance Copper Alloy Materials and Processing, Ministry of Education, Hefei University of Technology, Hefei 230009, Anhui, China

[6] Neutron Scattering Division, Oak Ridge National Laboratory, Oak Ridge, Tennessee 37831, USA

[7] Materials Science and Devices Institute, Suzhou University of Science and Technology, Suzhou, Jiangsu 215009, China

[8] CAS Key Laboratory of Design and Assembly of Functional Nanostructures, Fuzhou, 360002, China

[9] Brookhaven National Laboratory, National Synchrotron Light Source II, Upton, New York, USA

[10] Department of Materials Science and Engineering, University of Texas at Dallas, Richardson, Texas, USA

[11] School of Materials Science and Engineering, Anhui University, Hefei, 230601, China

*2021800026@hfut.edu.cn, *hfxiang@hfut.edu.cn

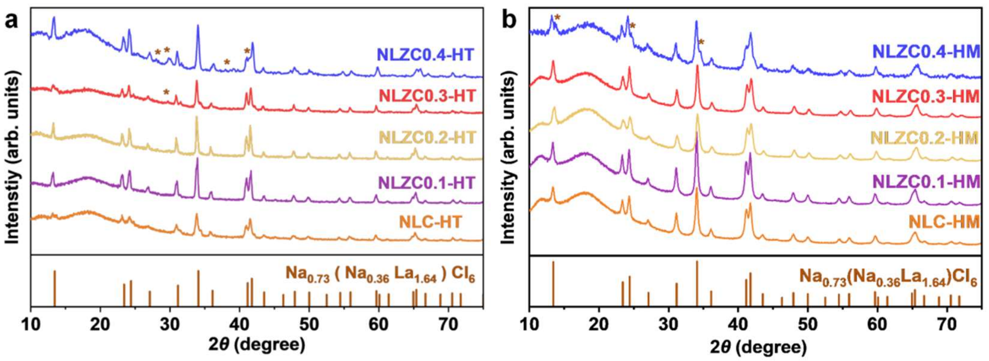

Supplementary Figure 1 XRD patterns of NLZC*x*-HT (a) with high temperature sintering and NLZC*x*-HM (b) after ball milling.

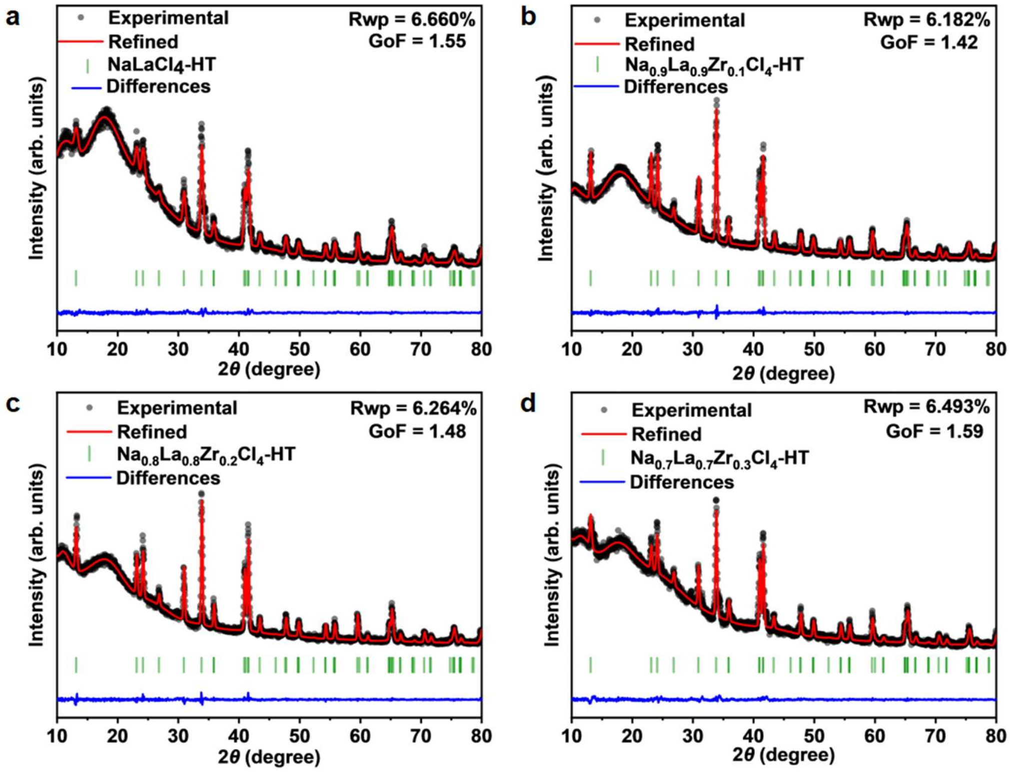

Supplementary Figure 2 Rietveld refinements of $NaLaCl_4$-HT (a), $Na_{0.9}La_{0.9}Zr_{0.1}Cl_4$-HT (b), $Na_{0.8}La_{0.8}Zr_{0.2}Cl_4$-HT (c), $Na_{0.7}La_{0.7}Zr_{0.3}Cl_4$-HT (d).

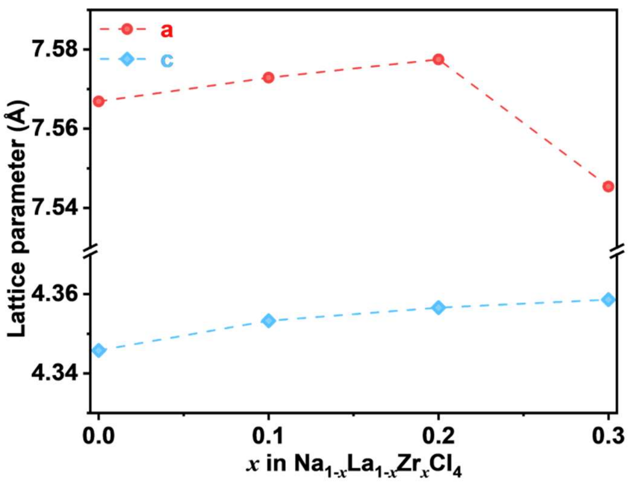

Supplementary Figure 3 Lattice parameters of $Na_{1-x}La_{1-x}Zr_xCl_4$-HT obtained from XRD refinement

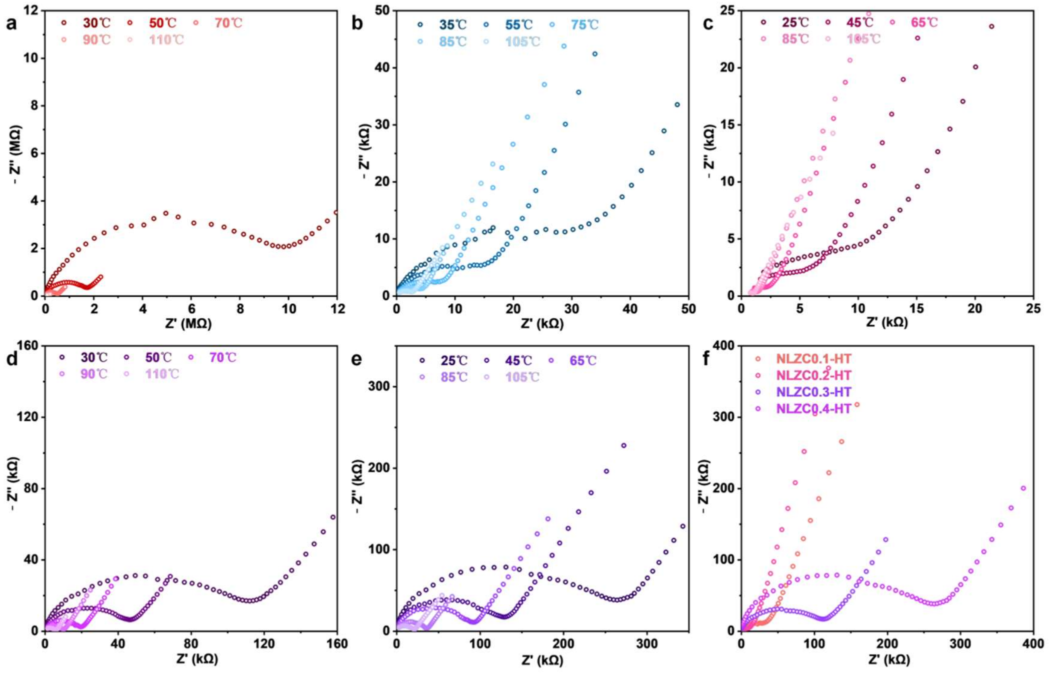

Supplementary Figure 4 Variable temperature Nyquist plots of NLZC*x*-HT with high temperature sintering: a) NLC-HT. b) NLZC0.1-HT. c) NLZC0.2-HT. d) NLZC0.3-HT. e) NLZC0.4-HT. Nyquist plots of NLZC*x*-HT at room temperature (f).

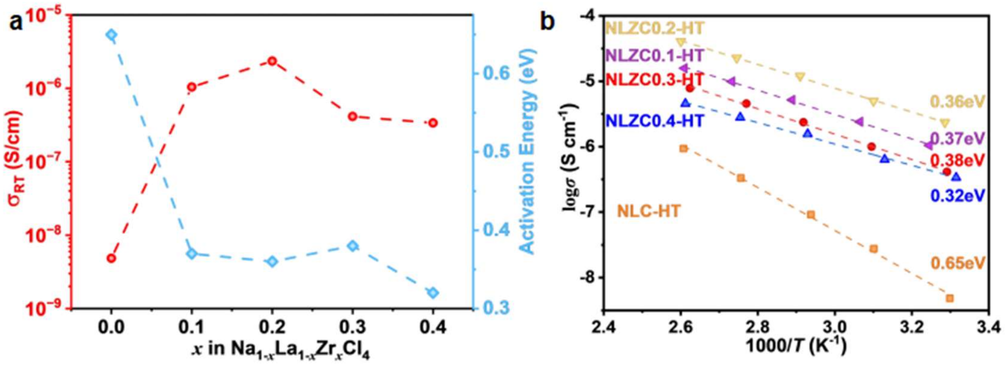

Supplementary Figure 5 a) Ionic conductivity and activation energy and b) Arrhenius conductivity plots of NLZCx-HT ($0 \leq x \leq 0.4$) with high temperature sintering.

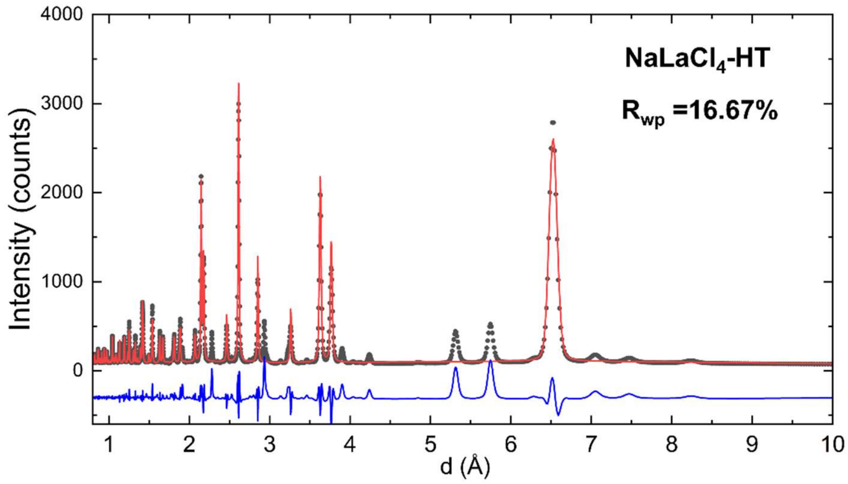

Supplementary Figure 6 Synchrotron XRD refinements of $NaLaCl_4$-HT

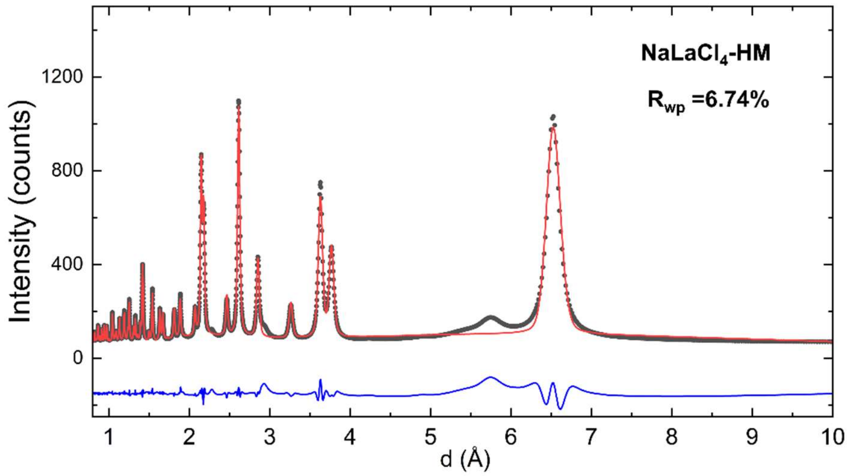

Supplementary Figure 7 Synchrotron XRD refinements of $NaLaCl_4$-HM

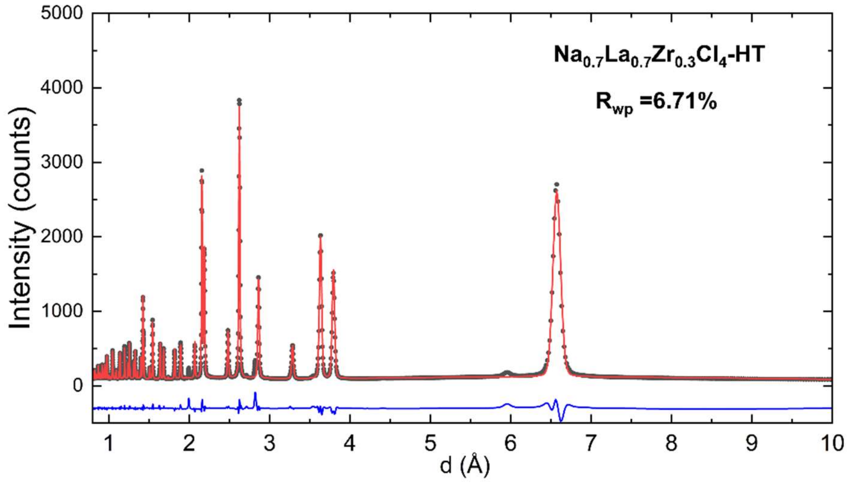

Supplementary Figure 8 Synchrotron XRD refinements of $Na_{0.7}La_{0.7}Zr_{0.3}Cl_4$-HT

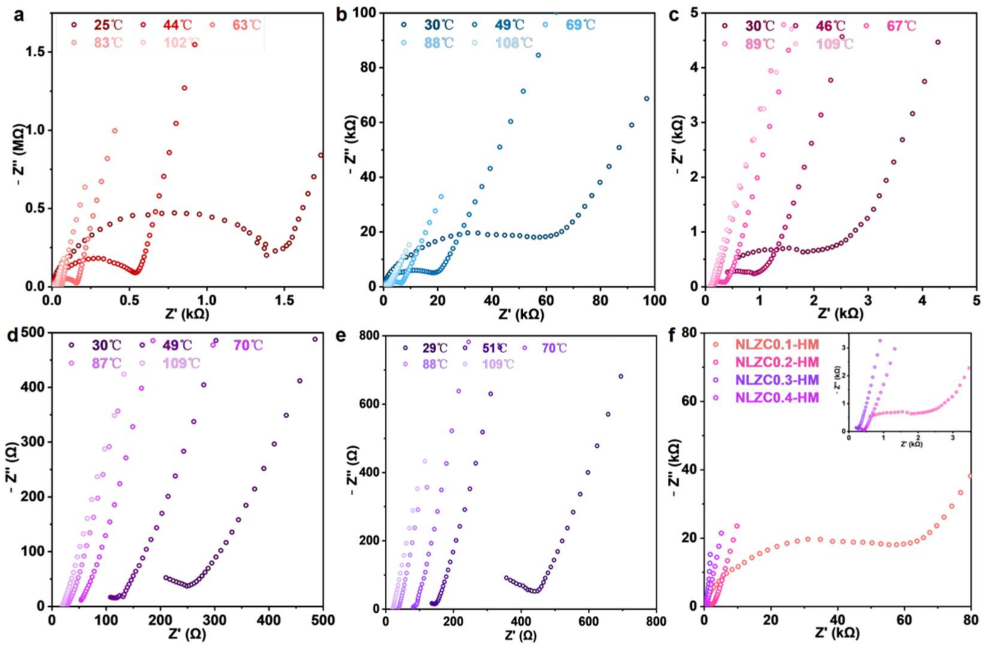

Supplementary Figure 9 Variable temperature Nyquist plots of NLZC*x*-HM: a) NLC-HM. b) NLZC0.1-HM. c) NLZC0.2-HM. d) NLZC0.3-HM. e) NLZC0.4-HM. Nyquist plots of NLZC*x*-HM at room temperature (f).

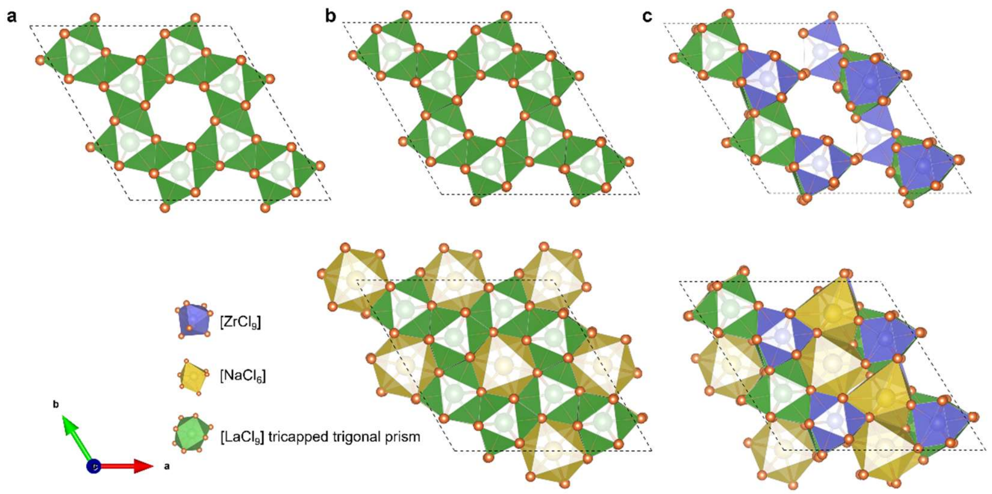

Supplementary Figure 10 Polyhedral style structure of (a) $LaCl_3$, (b) NLC and (c) NLZC. The $[NaCl_6]$ tricapped trigonal prism is eliminated in the upper part to better display the tunnel of the structure.

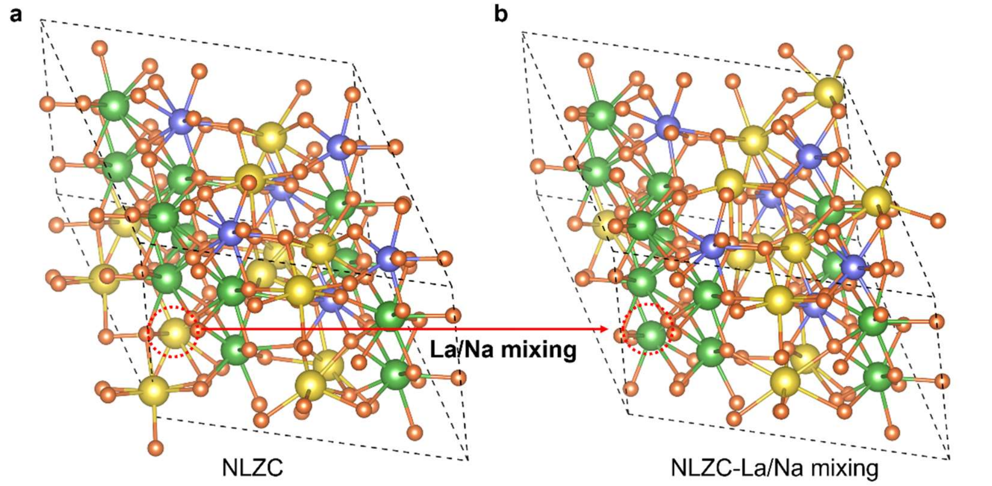

Supplementary Figure 11. (a) NLZC model and (b) NLZC-La/Na mixing model. The model of NLZC-La/Na mixing is selected from six mixing structures based on energy. (The green ball: La ions; The yellow ball: Na ions; The blue ball: Zr ions）

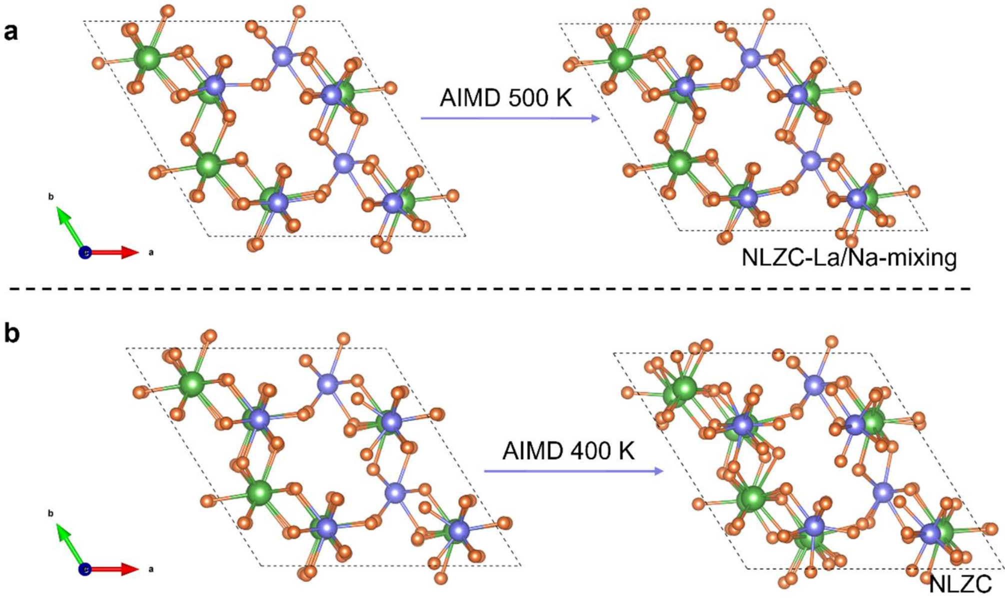

Supplementary Figure 12. (a) The structural changes before and after the simulation of NLZC-La/Na-mixing in 500 K AIMD and (b) The structural changes before and after the simulation of NLZC under 400K. (The green ball: La ions; The blue ball: Zr ions）

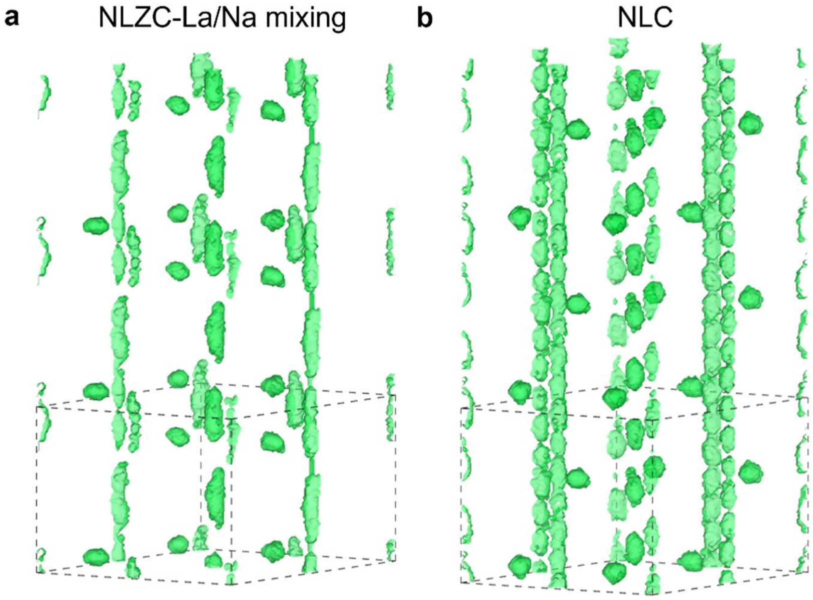

Supplementary Figure 13 Na^+ probability density, represented by green isosurfaces from AIMD simulations at 300 K of (a) NLZC-La/Na mixing and (b) NLC.

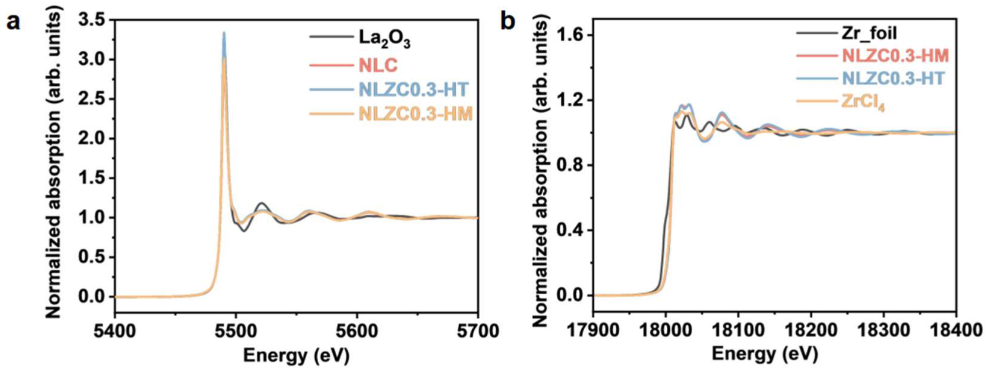

Supplementary Figure 14 (a)La L_3-edge and (b)Zr *K*-edge XANES spectra for the $Na_{0.7}La_{0.7}Zr_{0.3}Cl_4$ and $NaLaCl_4$

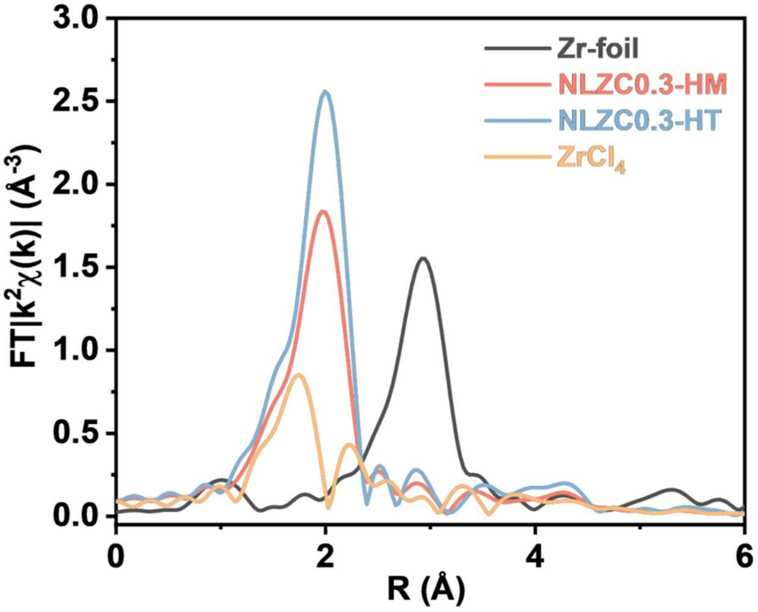

Supplementary Figure 15 R-space of Zr *K*-edge XANES spectra for $Na_{0.7}La_{0.7}Zr_{0.3}Cl_4$

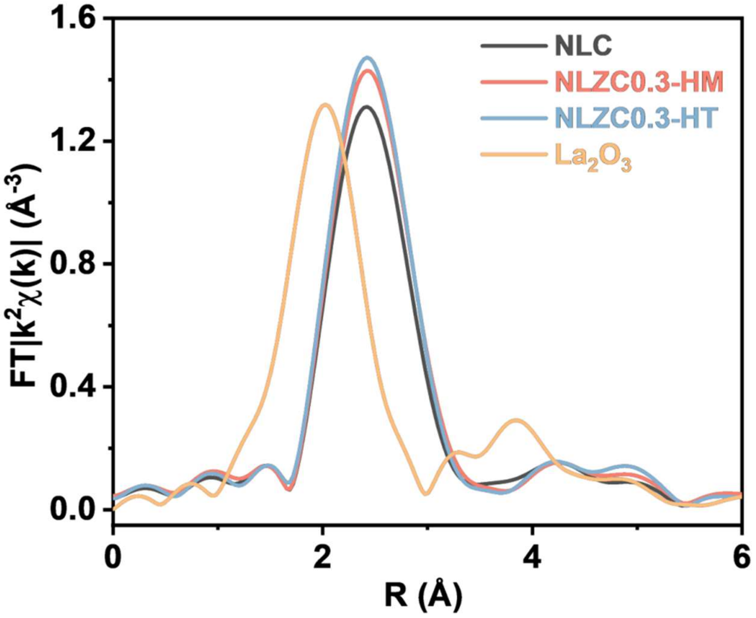

Supplementary Figure 16 R space of La L_3-edge XANES spectra for $Na_{0.7}La_{0.7}Zr_{0.3}Cl_4$ and $NaLaCl_4$

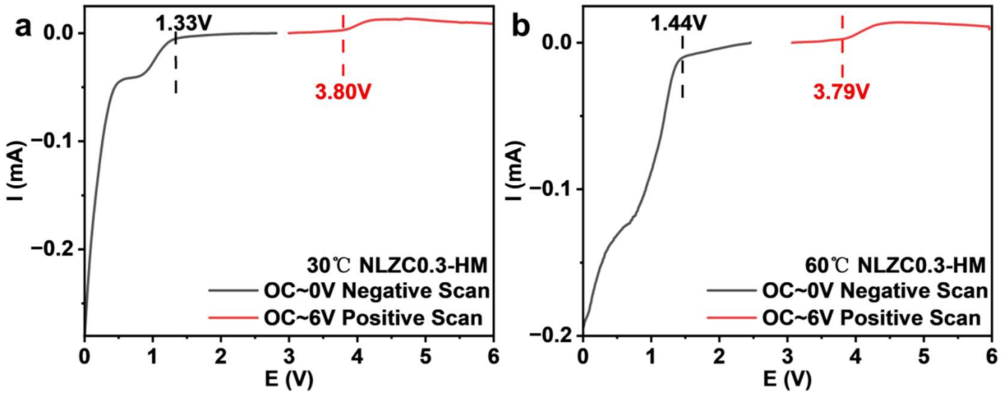

Supplementary Figure 17 Linear scanning voltammetry of NLZC0.3-HM at 0.1 mV s^{-1} and (a)30°C, and (b) 60℃

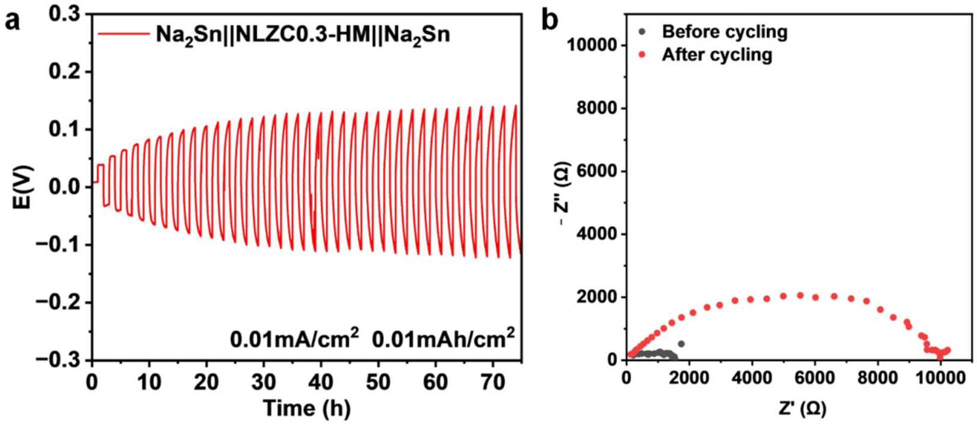

Supplementary Figure 18 (a) Galvanostatic cycling of the Na_2Sn||NLZC0.3-HM||Na_2Sn symmetric cells at 30℃. (b) Nyquist plots of Na_2Sn||NLZC0.3-HM||Na_2Sn before and after cycling.

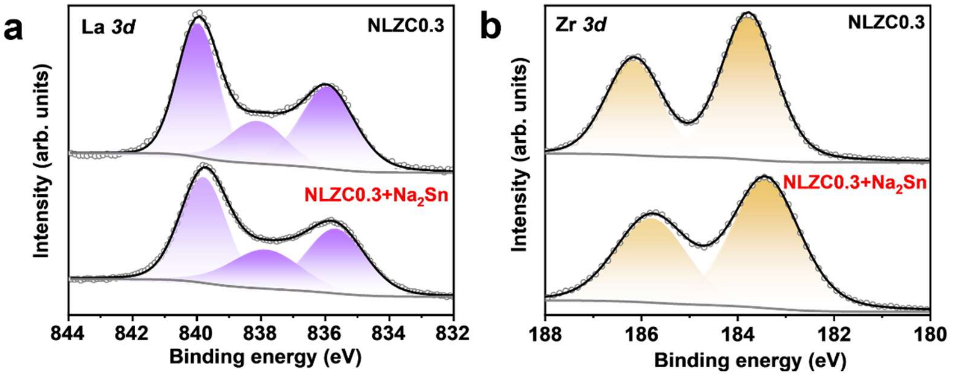

Supplementary Figure 19 (a) La *3d* and (b) Zr *3d* X-ray photoelectron spectroscopy (XPS) spectra of NLZC0.3, and NLZC0.3-HM+Na_2Sn.

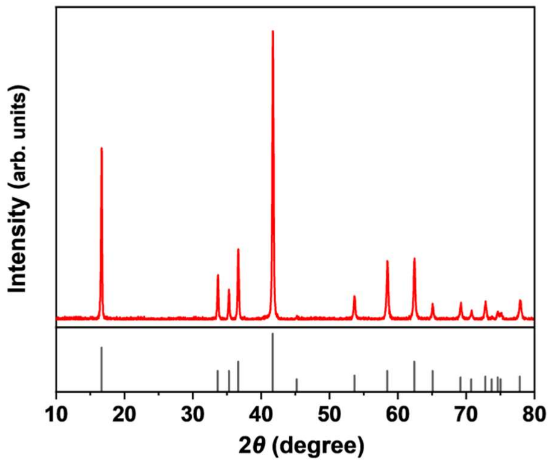

Supplementary Figure 20 XRD pattern of $NaCrO_2$

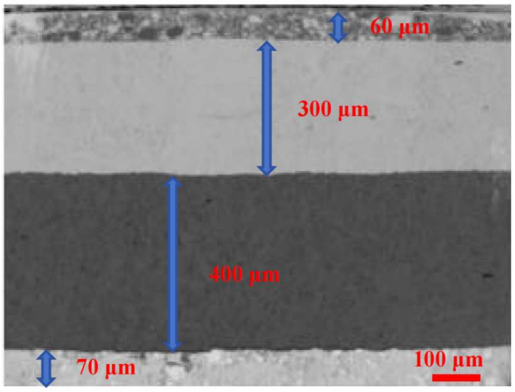

Supplementary Figure 21 Cross section SEM of the ASSB: these are $NaCrO_2$ composite (60 µm), NLZC0.3-HM (300 µm), Na_3PS_4 (400 µm), and Na_2Sn (70 µm) from the top to the bottom.

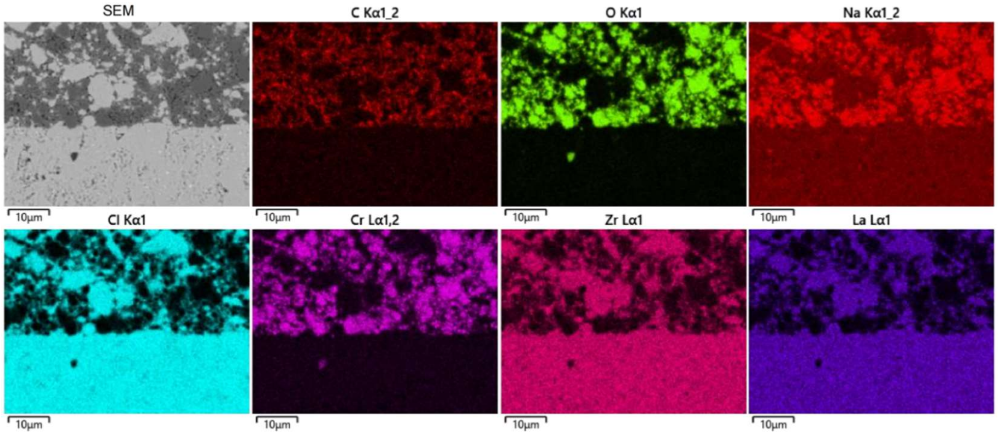

Supplementary Figure 22 SEM and EDS of the $NaCrO_2$ composite cathode layer.

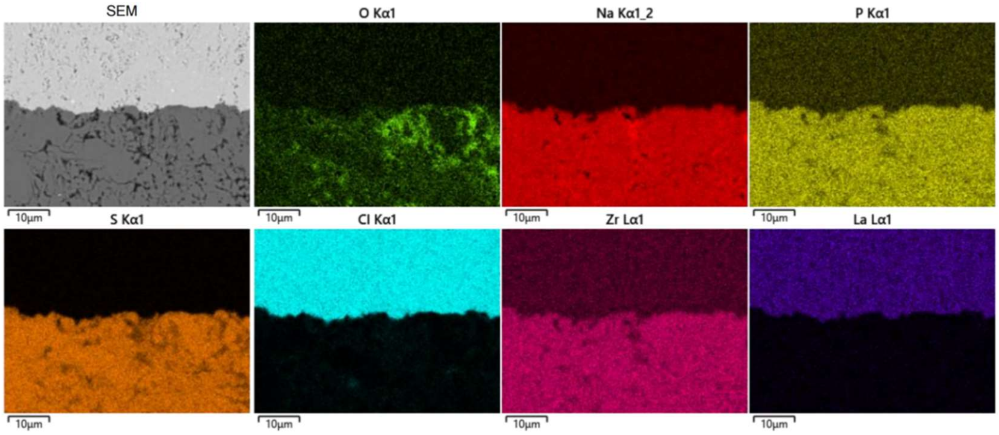

Supplementary Figure 23 SEM and EDS of the NLZC0.3-HM SSE layer.

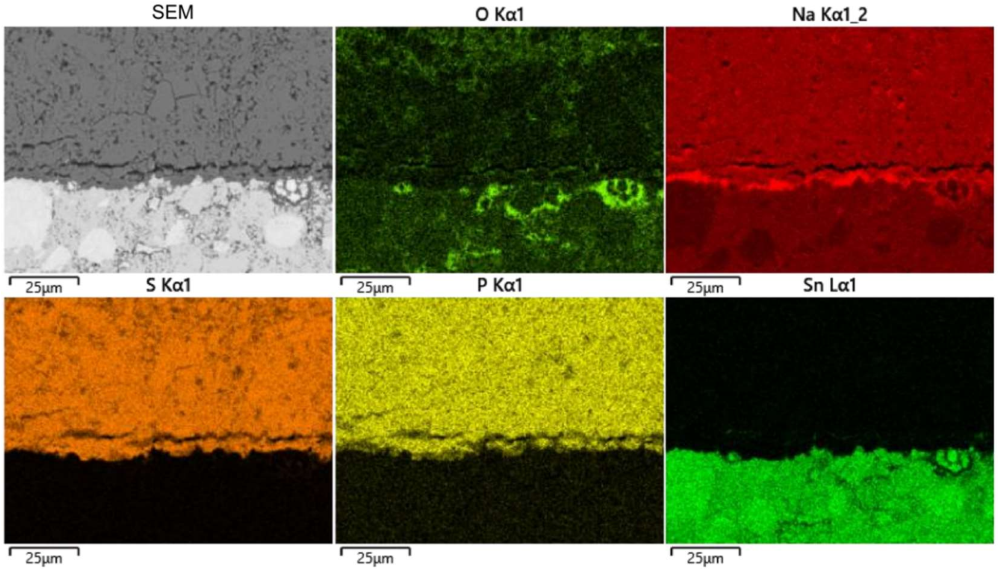

Supplementary Figure 24 SEM and EDS of the Na_3PS_4 SSE layer.

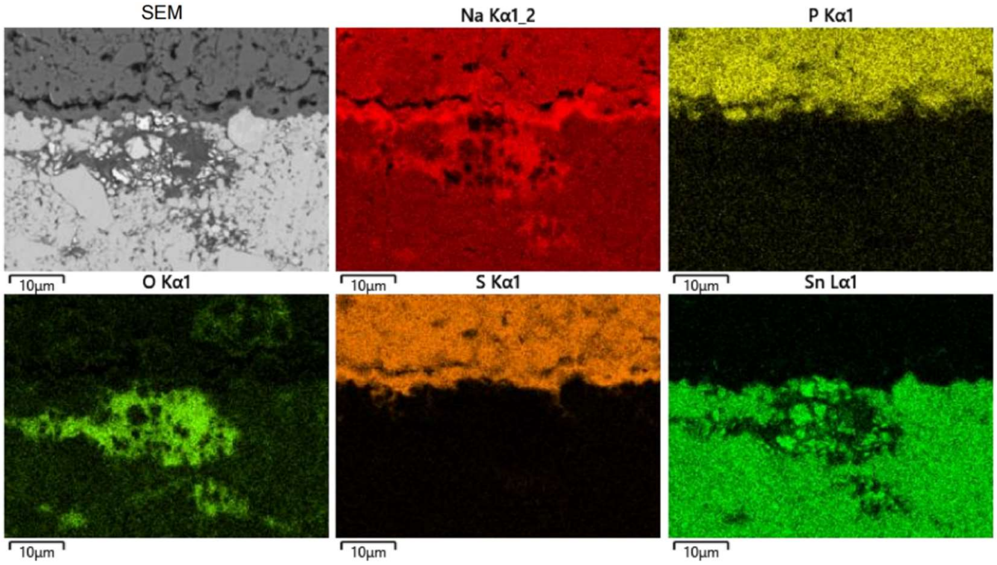

Supplementary Figure 25 SEM and EDS of the Na_2Sn anode layer.

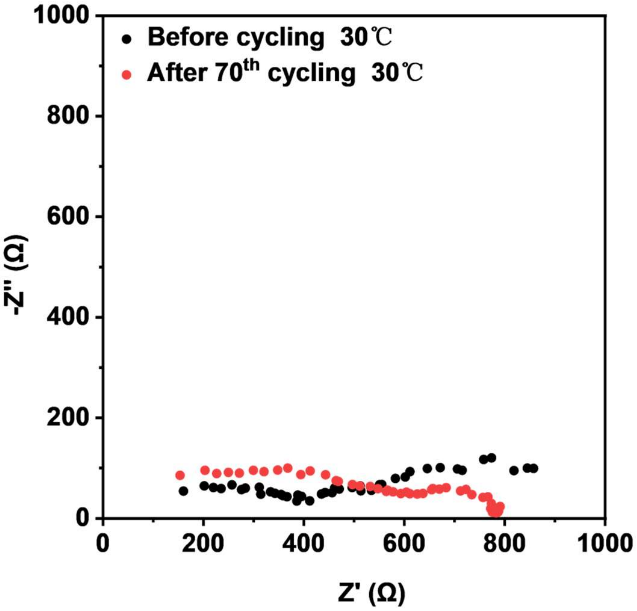

Supplementary Figure 26 Nyquist plots of $NaCrO_2$||NLZC0.3-HM||Na_3PS_4||Na_2Sn before and after cycling.

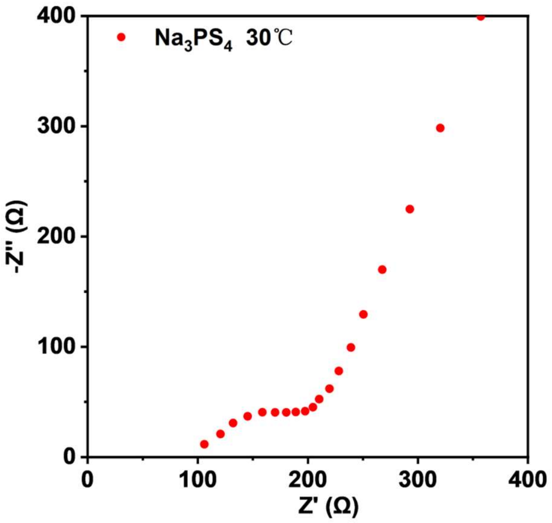

Supplementary Figure 27 Nyquist plots of Na_3PS_4 (1 mm in thickness).

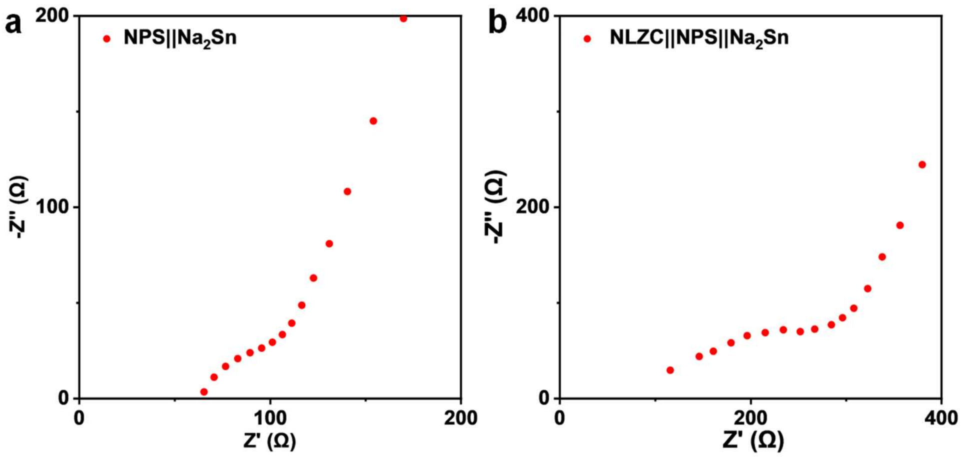

Supplementary Figure 28 Nyquist plots of Na_2Sn and Na_3PS_4 two layers (a), Na_2Sn, Na_3PS_4 and NLZC0.3-HM three layers (b).

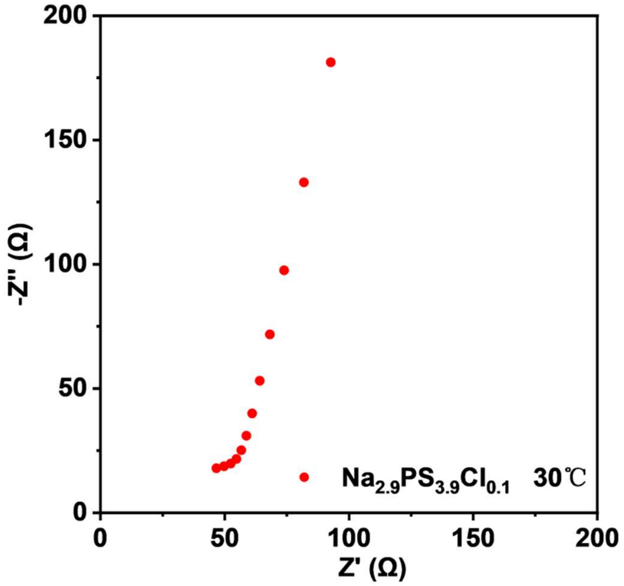

Supplementary Figure 29 Nyquist plots of $Na_{2.9}PS_{3.9}Cl_{0.1}$.

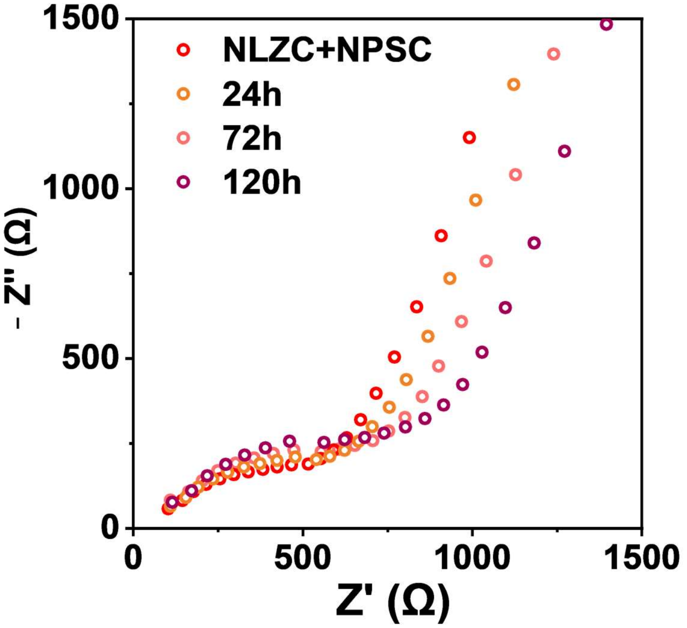

Supplementary Figure 30 Nyquist plots of NLZC0.3-HM and $Na_{2.9}PS_{3.9}Cl_{0.1}$ double layer SSE.

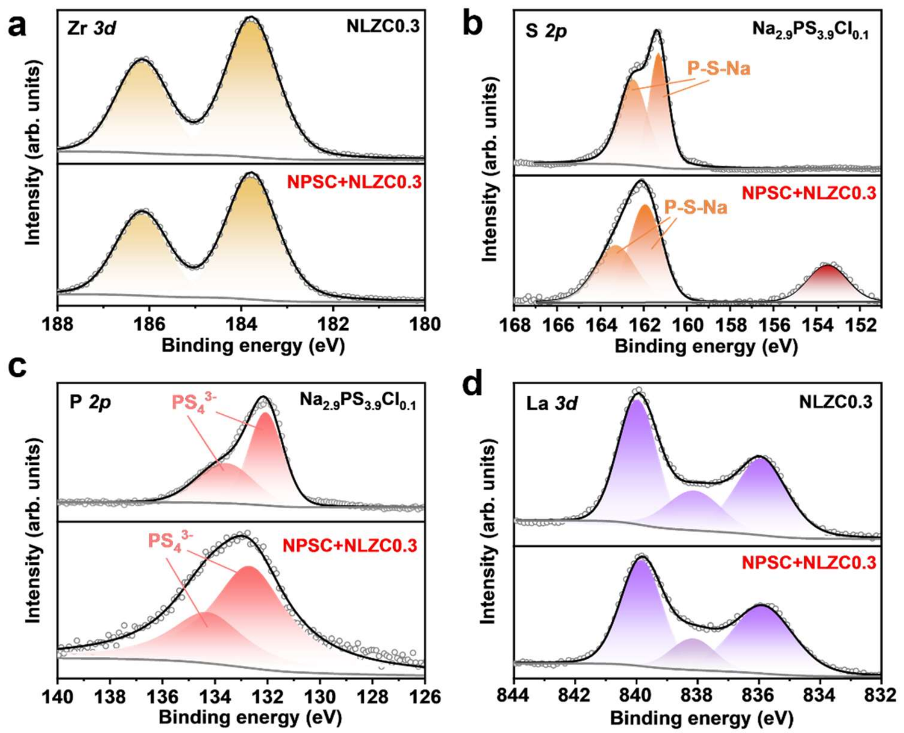

Supplementary Figure 31 (a) Zr *3d*, (b) S *2p*, (c) P *3d* and (d) La *3d* X-ray photoelectron spectroscopy (XPS) spectra of NLZC0.3, $Na_{2.9}PS_{3.9}Cl_{0.1}$ and NLZC0.3-HM+NPSC.

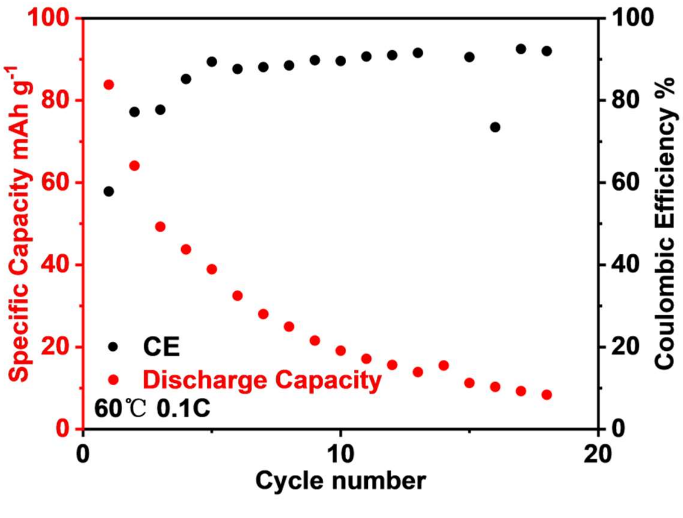

Supplementary Figure 32 Cycle performance of ASSB using $Na_{2.9}PS_{3.9}Cl_{0.1}$ SSE.

Supplementary Table 1 XRD refinement results of $NaLaCl_4$-HT

Component: $NaLaCl_4$-HT

Space group: $P6_3/m$

Lattice Parameters: $a = b$ =7.5669(9) Å, c = 4.3458(5) Å, V = 215.50(2) $Å^3$

$\alpha = \beta = 90°, \gamma = 120°$

Atom	x	y	z	Wyckoff	Occupancy	Uiso
Na1	0	0	0	2b	0.534	0.3419
Na2	0.33333	0.66667	0.25000	2c	0.233	0.0035(3)
La1	0.33333	0.66667	0.25000	2c	0.767	0.0035(3)
Cl1	0.38642	0.30207	0.25000	6h	1.000	0.0529(14)

R_{wp} = 6.660 %   GOF = 1.55 %

Supplementary Table 2 XRD refinement results of $Na_{0.9}La_{0.9}Zr_{0.1}Cl_4$-HT

Component: $Na_{0.9}La_{0.9}Zr_{0.1}Cl_4$-HT

Space group: $P6_3/m$

Lattice Parameters: $a = b$ = 7.5729(8) Å, c = 4.3533 Å, V = 216.21(A) $Å^3$

$\alpha = \beta = 90°, \gamma = 120°$

Atom	x	y	z	Wyckoff	Occupancy	Uiso
Na1	0	0	0	2b	0.331	0.3419
Na2	0.33333	0.66667	0.25000	2c	0.359	0.0035(3)
La1	0.33333	0.66667	0.25000	2c	0.559	0.0035(3)
La2	0.00000	0.00000	0.00000	2b	0.131	0.3419
Zr1	0.33333	0.66667	0.25000	2c	0.082	0.0035(3)
Cl1	0.38642	0.30207	0.25000	6h	1.000	0.0529(14)

R_{wp} = 6.182 %   GOF= 1.42 %

Supplementary Table 3 XRD refinement results of $Na_{0.8}La_{0.8}Zr_{0.2}Cl_4$-HT

Component：$Na_{0.8}La_{0.8}Zr_{0.2}Cl_4$-HT

Space group：*P*6₃/*m*

Lattice Parameters：*a* = *b* =7.5775(6) Å, c = 4.3566(7) Å, V = 216.64(3) Å^3

α = *β* = 90°, *γ* = 120°

Atom	x	y	z	Wyckoff	Occupancy	Uiso
Na1	0	0	0	2b	0.282	0.3419
Na2	0.33333	0.66667	0.25000	2c	0.331	0.0035(3)
La1	0.33333	0.66667	0.25000	2c	0.505	0.0035(3)
La2	0.00000	0.00000	0.00000	2b	0.108	0.3419
Zr1	0.33333	0.66667	0.25000	2c	0.164	0.0035(3)
Cl1	0.38642	0.30207	0.25000	6h	1.000	0.0529(14)
R_{wp} = 6.264% GOF= 1.48 %						

Supplementary Table 4 XRD refinement results of $Na_{0.7}La_{0.7}Zr_{0.3}Cl_4$-HT

Component：$Na_{0.7}La_{0.7}Zr_{0.3}Cl_4$-HT

Space group：*P*6₃/*m*

Lattice Parameters：*a* = *b* =7.5454(9) Å, *c* =4.3586(4) Å, V = 214.90(9) Å^3

α = *β* = 90°, *γ* = 120°

Atom	x	y	z	Wyckoff	Occupancy	Uiso
Na1	0	0	0	2b	0.266	0.3419
Na2	0.33333	0.66667	0.25000	2c	0.259	0.0035(3)
La1	0.33333	0.66667	0.25000	2c	0.516	0.0035(3)
La2	0.00000	0.00000	0.00000	2b	0.009	0.3419
Zr1	0.33333	0.66667	0.25000	2c	0.225	0.0035(3)
Cl1	0.38642	0.30207	0.25000	6h	1.000	0.0529(14)
R_{wp} = 6.493% GOF= 1.59 %						

Supplementary Table 5 XRD refinement results of $NaLaCl_4$-HM

**Component:** $NaLaCl_4$-HM						
**Space group:** $P6_3/m$						
**Lattice Parameters:** $a = b = 7.5646(16)$ Å, $c = 4.3717(5)$ Å, V = 216.65(6) $Å^3$; $\alpha = \beta = 90°$, $\gamma = 120°$						
Atom	x	y	z	Wyckoff	Occupancy	Uiso
Na1	0	0	0	2b	0.534	0.3419
Na2	0.33333	0.66667	0.25000	2c	0.233	0.0035(3)
La1	0.33333	0.66667	0.25000	2c	0.767	0.0035(3)
Cl1	0.38642	0.30207	0.25000	6h	1.000	0.0529(14)
R_{wp} = 3.898 % GoF = 1.79 %						

Supplementary Table 6 XRD refinement results of $Na_{0.9}La_{0.9}Zr_{0.1}Cl_4$-HM

**Component:** $Na_{0.9}La_{0.9}Zr_{0.1}Cl_4$-HM						
**Space group:** $P6_3/m$						
**Lattice Parameters:** $a = b = 7.5699(17)$ Å, $c = 4.3717(5)$ Å, V = 216.95(7) $Å^3$; $\alpha = \beta = 90°$, $\gamma = 120°$						
Atom	x	y	z	Wyckoff	Occupancy	Uiso
Na1	0	0	0	2b	0.391	0.3419
Na2	0.33333	0.66667	0.25000	2c	0.399	0.0035(3)
La1	0.33333	0.66667	0.25000	2c	0.619	0.0035(3)
La2	0.00000	0.00000	0.00000	2b	0.071	0.3419
Zr1	0.33333	0.66667	0.25000	2c	0.082	0.0035(3)
Cl1	0.38642	0.30207	0.25000	6h	1.000	0.0529(14)
R_{wp} = 3.497% GOF = 1.63 %						

Supplementary Table 7 XRD refinement results of $Na_{0.8}La_{0.8}Zr_{0.2}Cl_4$-HM

**Component**：$Na_{0.8}La_{0.8}Zr_{0.2}Cl_4$-HM<br>**Space group**：*P6₃/m*<br>**Lattice Parameters**：*a* = *b* = 7.5702(17) Å, *c* = 4.3718(5) Å, V = 216.97(7) Å^3<br>*α* = *β* = 90°, *γ* = 120°						
Atom	x	y	z	Wyckoff	Occupancy	Uiso
Na1	0	0	0	2b	0.330	0.3419
Na2	0.33333	0.66667	0.25000	2c	0.283	0.0035(3)
La1	0.33333	0.66667	0.25000	2c	0.553	0.0035(3)
La2	0.00000	0.00000	0.00000	2b	0.061	0.3419
Zr1	0.33333	0.66667	0.25000	2c	0.164	0.0035(3)
Cl1	0.38642	0.30207	0.25000	6h	1.000	0.0529(14)
R_{wp} = 3.522% GOF = 1.65 %						

Supplementary Table 8 XRD refinement results of $Na_{0.7}La_{0.7}Zr_{0.3}Cl_4$-HM

**Component**：$Na_{0.7}La_{0.7}Zr_{0.3}Cl_4$-HM<br>**Space group**：*P6₃/m*<br>**Lattice Parameters**：*a* = *b* = 7.6048(21) Å, *c* = 4.3999(7) Å, V = 220.37(10) Å^3<br>*α* = *β* = 90°, *γ* = 120°						
Atom	x	y	z	Wyckoff	Occupancy	Uiso
Na1	0	0	0	2b	0.281	0.3419
Na2	0.33333	0.66667	0.25000	2c	0.244	0.0035(3)
La1	0.33333	0.66667	0.25000	2c	0.511	0.0035(3)
La2	0.00000	0.00000	0.00000	2b	0.061	0.3419
Zr1	0.33333	0.66667	0.25000	2c	0.245	0.0035(3)
Cl1	0.38642	0.30207	0.25000	6h	1.000	0.0529(14)
R_{wp} = 3.583% GOF = 1.63 %						

Supplementary Table 9 Synchrotron XRD refinement results of $NaLaCl_4$-HT

**Component:** $NaLaCl_4$-HT					
**Space group:** *P6₃/m*					
**Lattice Parameters:** $a = b = 7.530$ Å, $c = 4.365$ Å, V = 214.36 Å^3					
$\alpha = \beta = 90°$, $\gamma = 120°$					
Atom	x	y	z	Wyckoff	Occupancy
Na1	0	0	0	2b	0.16
Na2	0.28555	0.68559	0.25000	6h	0.07
La1	0.33333	0.66667	0.25000	2c	0.87
Cl1	0.08510	0.38930	0.75000	6h	1

Supplementary Table 10 Synchrotron XRD refinement results of $NaLaCl_4$-HM

**Component:** $NaLaCl_4$-HM					
**Space group:** $P6_3/m$					
**Lattice Parameters:** $a = b = 7.534$ Å, $c = 4.368$ Å, V = 214.75 Å^3					
$\alpha = \beta = 90°$, $\gamma = 120°$					
Atom	x	y	z	Wyckoff	Occupancy
Na1	0	0	0	2b	0.251
Na2	0.28140	0.66100	0.25000	6h	0.034
La1	0.33333	0.66667	0.25000	2c	0.882
Cl1	0.08290	0.38590	0.75000	6h	1

Supplementary Table 11 Synchrotron XRD refinement results of $Na_{0.7}La_{0.7}Zr_{0.3}Cl_4$-HT

Component: $Na_{0.7}La_{0.7}Zr_{0.3}Cl_4$-HT

Space group: *P6₃/m*

Lattice Parameters: $a = b = 7.583$ Å, $c = 4.359$ Å, V = 217.09 Å^3

$\alpha = \beta = 90°$, $\gamma = 120°$

Atom	x	y	z	Wyckoff	Occupancy
Na1	0	0	0	2b	0.461
Zr1	0.33333	0.66667	0.25000	2c	0.104
La1	0.33333	0.66667	0.25000	2c	0.826
Cl1	0.08333	0.38550	0.75000	6h	1

Supplementary Table 12 Fitting results from EXAFS

Sample	R-factor	Path	N	R[Å]	ΔE_0 [eV]	$\sigma^2[10^{-3}$Å$^2]$
Zr foil	0.0007	Zr-Zr	6*	3.16	5.7	3.0
		Zr-Zr	6*	3.27		3.0
$ZrCl_4$	0.0076	Zr-Cl	2*	2.31	-1.52	1.3
		Zr-Cl	2*	2.47		2.8
		Zr-Cl	2*	2.63		5.2
Zr-NLZC0.3-HT	0.0024	Zr-Cl	7.3	2.48	1.26	3.7
Zr-NLZC0.3-HM	0.0031	Zr-Cl	6.9	2.48	0.82	6.5
La_2O_3	0.0084	La-O	6*	2.53	15.1	14.0
La-NLC	0.0029	La-Cl	8.2	2.93	17.5	15.9
La-NLZC0.3-HT	0.0021	La-Cl	8.9	2.94	17.7	15.0
La-NLZC0.3-HM	0.0032	La-Cl	8.8	2.94	18.2	15.5

* is fixed parameter.
